# Clozapine levels and therapeutic response: using individual patient meta-analysis data for a ROC Curve analysis

**DOI:** 10.1192/j.eurpsy.2023.587

**Published:** 2023-07-19

**Authors:** K. Northwood, E. Pearson, E. Wagner, N. Warren, D. Siskind

**Affiliations:** ^1^University of Queensland, Woolloongabba; ^2^Flinders University, Adelaide, Australia; ^3^LMU, Munich, Germany

## Abstract

**Introduction:**

Clozapine has been well established as the most efficacious medication for treatment refractory schizophrenia. Optimising the benefit during clozapine trial is an important clinical consideration. Therapeutic drug monitoring of clozapine plasma or serum levels has formed a critical part of this. Though there is no agreed standardised therapeutic range, advice traditionally recommends a clozapine level of >350ng/mL in order to effect best response. Most studies analysing the relationship between treatment response and clozapine level are older, have small sample sizes, and do not consider whether additional factors might assist in determining optimal clozapine level for response.

**Objectives:**

We conducted a systematic review of PubMed, PsycInfo and Embase for studies that provided individual participant level data on clozapine levels and response.

**Methods:**

This data was analysed using Receiver Operating Characteristic (ROC) curves to determine the prediction performance of serum clozapine levels for treatment response.

**Results:**

We were able to include data on 294 individual participants. ROC analysis yielded an area under the curve (AUC) of 0.612. The clozapine level at the optimal Youden index was 372ng/mL, and at this level there was response sensitivity of 57.3%, and specificity of 65.7%. The interquartile range for treatment response was 223ng/mL – 558ng/mL. There was no improvement in ROC performance with mixed models including patient sex, age or length of trial.

**Conclusions:**

Clozapine dose should be optimised based on clozapine therapeutic levels. We found that a range between 250 – 550ng/mL could be recommended, while noting that a level of >350ng/mL is most optimal for response.

**Disclosure of Interest:**

None Declared

